# Emotional impact on children during home confinement in Spain

**DOI:** 10.3389/fpubh.2022.969922

**Published:** 2022-10-14

**Authors:** Francisco Sánchez-Ferrer, Evelyn Cervantes-García, César Gavilán-Martín, José Antonio Quesada, Ernesto Cortes-Castell, Ana Pilar Nso-Roca

**Affiliations:** ^1^San Juan de Alicante University Hospital, Sant Joan d'Alacant, Spain; ^2^Department of Pharmacology, Pediatrics and Organic Chemistry, Miguel Hernández University Medical School, Miguel Hernández University of Elche, Sant Joan d'Alacant, Spain; ^3^Department of Clinical Medicine, Miguel Hernández University Medical School, Miguel Hernández University of Elche, Sant Joan d'Alacant, Spain

**Keywords:** confinement, COVID-19, emotional impact, sadness, irritability, children

## Abstract

**Introduction:**

The COVID-19 pandemic has brought about important changes. On March 14, 2020, a strict home confinement was decreed in Spain. Children did not attend school and were not allowed to leave their homes. The aim of this study was to determine the emotional state of these children, as well as associated factors.

**Material and methods:**

A cross-sectional descriptive study was conducted using an online questionnaire sent by cell phone. This survey includes sociodemographic items and questions concerning the emotional impact of the lockdown. With the questions on emotions, two categories of emotional state were established with the variables fear, irritability, sadness and somatization: those who were less or more emotionally affected. A multivariate logistic model was used to estimate the associations between the variables.

**Results:**

A total of 3,890 responses were obtained. The mean age of the children was 6.78 years (range 0 to 16). A score indicating poor emotional state was reported by 40.12%. The multivariate logistic model for poor emotional state was directly associated with having less appetite, sleep disturbances, and with parents' beliefs that their child will have difficulties returning to normal life after lockdown. A better emotional state was associated with being an only child, access to outdoor spaces at home, having pets, and parents informing their children about the pandemic using creative explanations.

**Conclusions:**

During strict home confinement, a considerable emotional impact was observed in children as described by their parents. Specific elements were associated with a better or poorer emotional state.

## Introduction

The outbreak of severe acute respiratory syndrome coronavirus 2 (SARS-CoV-2), which was first reported in Wuhan, China, in December 2019, had a tremendous impact initially in China and subsequently throughout the world. While most patients infected with SARS-CoV-2 had mild illness, about 5% of patients experienced severe lung injury or multi-organ dysfunction, resulting in a 1–4% case fatality rate (1).

Restrictive measures in the general population were necessary to reduce the rate of virus transmission. Quarantines and pandemic disasters have demonstrated negative psychological effects, including confusion, anger, and post-traumatic distress (2, 3).

During the first outbreak in Europe in March 2020, a strict lockdown took place in Spain with the population confined to their homes. Only essential work was permitted, and the population was allowed to leave their homes only for basic activities. In Spain, this strict lockdown was declared as of March 13, 2020. In addition to schools being closed, children were not allowed to leave their homes under any circumstances (except to go to the doctor or to accompany their parents for essential activities if they would have been left at home alone). As of April 26, children were allowed to leave their homes for a reduced period of time. Prior to COVID-19, a review in The Lancet on quarantines revealed their broad and potentially long-lasting negative psychological consequences (3). Another general population study compared mental health during the pandemic period and in the same time period 3 years prior. A clear psychological deterioration was found, with an increase in depression that was more pronounced in young adults (4).

Confinements have produced an emotional impact on the entire population (5) and children (6). The effects of the lockdown on emotional well-being have been perceived as negative (3, 7), with increased stress, anger, fear, and confusion (8). In children, it has also been shown that the COVID-19 pandemic has had a significant psychological impact (9). Children are frightened, nervous, sad, bored, and angry but also feel safe, calm, and happy to be with their families (10). Nonetheless, these negative feelings have been more prevalent and can affect the entire family (11). In adolescents, this has been associated with depression and anxiety (12).

During the confinement, daily routines have been altered, such as sleep habits (13, 14). These changes may have long-term emotional effects (15). Other habits, such as eating patterns, have also undergone changes in this period (16). In addition, activities that help improve the emotional health of young people, such as exercise (17), physiotherapy, relaxation, and academic performance, were restricted (18).

The emotional impact depends on many biological and sociodemographic factors that must be taken into account (19). The degree to which parents are affected also influences their children (20, 21). These effects on the family and children, as well as their needs during the first outbreak, were not taken into consideration (22, 23).

Studies on emotional health in children during the COVID-19 pandemic have been conducted mainly in the Chinese population (24). There are many methodological limitations in the studies carried out, such as small sample sizes or being performed after the period of confinement with the resulting recall bias, among other limitations (12).

The studies in children during this period of confinement are highly relevant because of the great emotional impact described and because they enable us to determine the sociodemographic characteristics of the children at greatest risk. In addition, there are variables that may involve a greater emotional impact on children, such as the direct or indirect consequences of COVID in the family (23), the presence of some type of disability (24) or having parents who are essential workers during the pandemic (25). On the other hand, variables such as having pets (26) or having outdoor access at home can be protective (17). This information is important in order to be able to implement measures to limit the effects of future confinement on the emotional health of children.

The aim of this study was to assess the degree of emotional impact on children as perceived by their parents during the strict lockdown and to identify the factors associated with emotional state.

We analyzed the most common sociodemographic variables, paying special attention to housing conditions (due to the situation of confinement) and examined several dimensions that may be associated with emotional state, such as communication with the children, parental perception of after effects, the effect on illnesses or the physical repercussions on the children.

## Materials and methods

### Participants

A cross-sectional descriptive study was carried out through a questionnaire that was sent by instant messaging via cell phone to parents for completion. There was no sample selection. The inclusion criteria were residing in Spain and agreeing to complete the questionnaire. The questionnaire was available from April 22, 2020 to April 26, 2020. A total of 3,890 questionnaires were collected during the study period.

A panel of local experts met to construct a specific questionnaire to study the effect of the pandemic on children. Given the exceptional nature of the situation, with strict confinement, the previously validated questionnaires were considered unsuitable. To construct the questionnaire and select the possible variables related to emotional states, previous questionnaires that assess fear, anxiety and sadness were consulted, such as the KIDSCREEN (27, 28), the Liebowitz social anxiety scale (29), the Spielberger State-Trait Anxiety Inventory (STAI) (30, 31) or the Emotional Eating Scale Adapted for Children and Adolescents (EES-C) (32). Given the relationship of this emotional situation with somatization (3, 33) it was also included as an emotional variable. The presence of somatization signs was also included in the measurements due to its relationship with the emotional state.

### Patient and public involvement

It was not appropriate or possible to involve patients or the public in the design, or conduct, or reporting, or dissemination plans of our research.

### Variables

The first part includes the following variables: Age (years), Sex (boy/girl), Autonomous community (Spanish province), Do you have other children at home? (yes/no), Number of children at home (number), Number of adults at home (number), Location of home (no answer/urban/rural), Size of home (no answer/less 60 m^2^/60–120 m^2^/more 120 m^2^), Outdoor space at home (no answer/yes/no), Average academic grade (no answer/excellence/very good/satisfactory/unsatisfactory), Educational support (yes/no), Parents are health sector workers (yes/no), Parents are law enforcement workers (yes/no), Parents are other essential workers (yes/no), Have had Covid-19 (yes/no), Pets in the home (yes/no), and Underlying disease (yes/no).

### Questionnaire

The questionnaire for families, developed by a local group of experts, was divided into 5 dimensions with several questions in each:

Communication: Do you feel that you have given your child age-appropriate information (in words he/she can understand) about what is happening? (yes/no/I am not sure), To what extent have you given information to your child? (honest including negative aspects/honest avoiding negative aspects/no information), How have you approached the information given to your child? (realistic information/information by embellishing or misrepresenting the negative aspects/creative information/no information).

Normality during and after the pandemic: Do you feel that your child has accepted and adapted to the current situation? (yes/no/I am not sure), Do you think your child might have trouble returning to “normal” daily activities? (yes/no/I am not sure).

Control of disease: Only answer this question if your child has a medical condition. How do you think the confinement has affected the condition? (negative/positive/no changes), During this period, did you need to consult a medical professional because you were concerned about any aspect of your child's health? (yes/no).

Non-emotional involvement: Do you feel that your child is having trouble falling asleep or is sleeping worse than usual? (yes/no/I am not sure), Do you think there have been changes in your child's appetite? (yes/no/I am not sure), Regarding nutrition, do you feel that there have been changes in the quality of the diet during this period? (improved/worse/no changes/I am not sure), With regard to the time spent in front of screens (video consoles, television, electronic tablets, cell phones, etc.), indicate the average time spent daily by your child (in relation to the current situation) (1/1–2/2–3/3–4/more than 4 h/does not use).

Emotional involvement: Do you think your child has ever felt sad (in relation to the current situation)? (yes/no/I am not sure), Do you think your child has ever felt afraid (in relation to the current situation)? (yes/no/I am not sure), Do you think your child is more irritable? Example: more temper tantrums, less obedient, more sensitive (yes/no/I am not sure), During the confinement period, has your child had symptoms such as headache, abdominal pain, musculoskeletal pain, tiredness, etc. for no apparent reason and without this type of pain being usual previously? (somatization) (yes/no/I am not sure).

As a response variable, the dimension of emotional state was constructed from 4 items (sadness, fear, irritability and somatization) in the following way: A value of 1 is given to the response “yes” and 0 to the response “no” in each of the items. The total score for the response variable is the sum of all the items, ranging between 0 and 4, with 4 being the worst emotional state. It was grouped as scores of 3–4 vs. 0–2.

### Statistical analysis

A descriptive analysis of all the variables was performed by calculating frequencies for qualitative variables and minimum, maximum, mean, and standard deviation for quantitative variables. The factors associated with emotional state and type of information given to the child were analyzed using contingency tables applying the chi-squared test for qualitative variables and comparison of the mean values for quantitative variables using Student's *t*-test.

To estimate the magnitude of the associations with poor emotional state, multivariate logistic models were fitted. The total sample without missing data in the variables (*n* = 1,501) was randomly split into a training sample and a test sample at a ratio of 2/3 and 1/3. The model was adjusted in the training sample to arrive at an optimal model, applying the Homer-Lemeshow calibration test. Odds ratios (OR) were estimated, together with their 95% confidence intervals. A stepwise variable selection procedure based on the Akaike information criterion was performed. A validation process was conducted on the test sample, calculating the area under the ROC curve and its 95% confidence interval, Alpha level 0.05. All analyses were performed using R version 4.0.2.

## Results

The general characteristics of the sample are shown in Table 1. Women made up 48.6% of the sample, with a mean age of 6.78 years. Most of the respondents were preschool-age (36.3%) and school-age children (51.3%). The sample was primarily urban (77.7%) but almost half of the sample owned a house with a garden. Concerning the emotional state of the children, 44.9% were afraid, 67.5% sad, 64.2% irritable, and 29.6% experienced somatization of an illness. According to the constructed variable “emotional state,” in 33.8% this was poor (presence of 3 or 4 variables) (abbreviated Table 1 and complete tables in Supplementary materials 1, 2).

**Table 1:** Values of the variables analyzed in the total sample (abbreviated table).

**Variable**	***N* (%); mean ±SD**
Mean age of children (years)	6.78 (3.24)
Sex (Male)	772 (51.4)
Only child	338 (22.5)
Mean number of children in the family	1.93 (0.78)
Mean number of adults	2.03 (0.48)
Urban housing	3,108 (85.6)
Living area > 120 m^2^	1,061 (29.2)
Home with garden	1,732 (47.7)
Pets	1,152 (39.9)
Chronic illness	422 (10.8)
Sleep (worse)	1,689 (44.7)
Good emotional state (0–2)	1,349 (59.9)

*N* = 1,501.

*Table of the complete description of all the variables analyzed in Supplementary materials 1, 2.

Certain variables were significantly associated (*p* < 0.05) with a poorer emotional state, such as not being an only child, having sleep disturbances, not having a terrace or garden, parents believing that they will have problems returning to normal life after the pandemic, and giving honest information about the situation were significantly related to a worse emotional state. These variables are shown in Table 2 (abbreviated Table 2 and complete tables in Supplementary materials 3, 4).

**Table 2:** Summarized bivariate analysis of the variables analyzed with respect to emotional state (good 0–2 negative emotions or bad 3–4 negative emotions, including sadness, fear, irritability, and physical symptoms)^**^.

		**0–2 negative emotions**		**3–4 negative emotions**		
		***n***	**%**	***n***	**%**	***p*-value**
Information adapted to the age of the children	I have tried to be honest with them	931	65.2%	496	34.8%	<0.001
	I have preferred not to give information	63	85.1%	11	14.9%	
Situation will normalize after the pandemic	Yes	922	72.3%	354	27.7%	<0.001
	No	72	32.0%	153	68.0%	
Only child	No	719	64.0%	405	36.0%	0.001
	Yes	275	72.9%	102	27.1%	
Type of information given to the children	Realistic information	695	65.2%	371	34.8%	<0.001
	Information misrepresenting the negative aspects	172	60.6%	112	39.4%	
	Creative explanations	62	83.8%	12	16.2%	
	No information	65	84.4%	12	15.6%	
Information adapted to the age of the children	Yes	931	65.2%	496	34.8%	<0.001
	No	63	85.1%	11	14.9%	
Sleep disturbances	Yes	284	47.9%	309	52.1%	<0.001
	No	710	78.2%	198	21.8%	
Return to activity after pandemic	Yes	261	51.3%	248	48.7%	<0.001
	No	733	73.9%	259	26.1%	
Outdoor access at home (garden or terrace)	Yes	501	71.9%	196	28.1%	<0.001
	No	405	60.7%	262	39.3%	

*N* = 1,501.

** Table of the complete bivariate analysis of all variables analyzed in Supplementary materials 3, 4.

The multivariate logistic model performed to explain poor emotional state is shown in Table 3. Receiving information through creative explanations (OR 0.22, CI 0.073–0.70), having a home with a garden (OR 0.578, CI 0.35–1.00), being an only child (OR 0.68, CI 0.45–0.98), children not asking about the pandemic situation (OR 0.34, CI 0.24–0.48) or not having sleep disturbances (OR 0.41, CI 0.30–0.57) are elements that were associated with a better emotional state In contrast, not having pets (OR 1.44, CI 1.04–1.97), having less appetite (OR 2.30, CI 1.45–3.65), or parents who believe that life will not return to normal after the pandemic (OR 2.72 CI 1.79–4.15) were associated with a poor emotional state.

**Table 3:** Multivariate logistic model for poor emotional state (presence of 3–4 negative items).

		**Betha**	OR^*^	**CI 95%**	***p*-value**
Type of information given to the child	Realistic information	0	1		
	Information misrepresenting the negative aspects	0.05372	1.055	(0.712–1.565)	0.789
	Creative explanations	−1.48621	0.226	(0.073–0.700)	0.009
	No information	−0.58049	0.560	(0.227–1.378)	0.206
Appetite	Has more	0	1		
	No change	−0.30185	0.739	(0.510–1.072)	0.111
	Has less	0.83570	2.306	(1.454–3.658)	0.001
Home with garden	No answer	0	1		
	Yes	−0.54739	0.578	(0.335–1.000)	0.049
	No	−0.10606	0.899	(0.525–1.542)	0.699
Sleep disturbance	No	−0.88301	0.414	(0.300–0.570)	<0.001
Parent believes situation will not normalize	No	1.00382	2.729	(1.791–4.157)	<0.001
Only child	Yes	−0.40418	0.668	(0.453–0.983)	0.040
Pets in the home	No	0.36440	1.440	(1.047–1.979)	0.024
Children ask about what is happening	No	−1.06831	0.344	(0.243–0.485)	<0.001
Return to activity	No	−0.43769	0.646	(0.464–0.898)	0.009
Age		0.04036	1.041	(0.986–1.100)	0.148
Sex (Female)		0.16531	1.180	(0.867–1.605)	0.292

Training sample *n* = 1,000; test sample *n* = 501; number of children with a poor emotional state (3–4 items) in the training sample = 347; area under the ROC curve in the test sample = 0.812 (95% CI 0.773–0.850). Homer-Lemeshow *p*-value = 0.713; accuracy rate in the test sample = 75.4%. Nagelkerke's R^2^ = 0.339. Likelihood ratio chi-squared test = 281.8 (*p* < 0.001).

* Model adjusted for control of disease.

The model has an area under the ROC curve in the test sample of 0.812 (95% CI: 0.773–0.850), good calibration (Homer-Lemeshow test: *p*-value 0.713), and a success rate in the test sample of 75.4% (95% CI: 0.773–0.850) (Table 3).

## Discussion

The results of this study reveal the considerable emotional impact on children during the lockdown, identifying factors associated with a poor emotional state. Giving children information using creative explanations, living in a home with a garden, being an only child, and having pets were factors associated with less emotional distress. Conversely, having less appetite, disturbed sleep, or a parent who believes that the situation will not return to normal after confinement were associated with a greater impact on the emotional state of the child.

The period of strict home confinement of children in Spain in the spring of 2020 was one of the longest experienced. Children were unable to leave their homes for 6 weeks.

We sought to quantify this impact by means of a questionnaire for parents, in which they assessed the emotional situation of their child as well as providing various medical and sociodemographic variables. The start date of the survey was just 4 days before the end of the lockdown (following 38 days without leaving the home and up to the time when the lockdown officially ended).

Emotional experience was subjectively assessed by the parents through the items concerning their child's perception of sadness, fear, irritability, and physical symptoms. These values were answered dichotomously (Yes or No). With these data, we defined the emotional state as “good” (score 0–2) or “poor” (score 3–4). The findings indicate that confinement had an important effect, with 40.1% having a poor emotional state, with feelings of sadness and irritability experienced by approximately two-thirds of the children. These data are data are consistent with the existing literature (8, 9), with no gender differences found.

The emotional impact of the COVID-19 lockdown has also been described in children, and in all cases the effects are similar to our results (10, 11, 21, 25, 26). These effects have also been studied in adolescents and adults (7) as well as in parents (20). Most of these studies show a negative emotional impact that is greater in the younger population (5) and relatively smaller at older ages.

Multivariate analysis indicated that having siblings in the home increased emotional risk during the pandemic and could be explained by greater parental stress and household turmoil (21). Decreased appetite and sleep disturbances were also associated with a poor emotional state (13, 14, 27). Both of these elements are known to affect emotional well-being (28).

Similarly, parents' beliefs that their child may not be able to return to normal life after the pandemic could be associated with greater emotional distress, since it is the parents themselves who value their children emotionally and are aware of the difficulties they may have readapting to normal life after the lockdown.

By contrast, the existence of a garden at home was linked to a better emotional state in children in that they can “leave” the house to take a walk or to be in the sun, both elements traditionally associated with happiness, as well as increased access to physical exercise (18). In addition, having a garden was associated with another key element for the protection of mental health, namely, having greater economic resources (19). It is of note that the size of the house showed no significant association.

The connection observed between children having a good emotional state and parents providing creative explanations about the coronavirus could be could be due to the age of these children. Younger children would normally be given imaginative stories, while older children are given factual information and would be more affected. This coincides with studies demonstrating a greater impact on older children. In our study, the mean age of the children who did not receive information was 2.26 years and those who did receive information was 6.76 years, with a significant difference (*p* < 0.001). Finally, having pets in the home was associated with a good emotional state. Research postulates that animals provide greater social competence (29), which would be helpful to children in this context. In our paper, the physical activity is not studied. It is interesting that in other studies this physical activity (34) does not affect the emotional state (30). On the other hand, Cognitive-Behavioral Therapy in adolescents with low academic performance decreased their stress (31).

Recognizing that confinement can have a detrimental effect on mental health, especially in certain conditions such as the ones described above, provides incentives for measures to limit this effect, such as increased communication with parents, information about the disease with age-appropriate information and, of course, early access to mental health services (32). Public health authorities must take into consideration these emotional effects, not prolonging the confinement or quarantine for longer than necessary (3).

Further research in this area can help to better understand the factors associated with home confinement and the degree of emotional impact on children, which can be instrumental in identifying those at higher risk and to implement interventions to enhance emotional well-being in this more vulnerable group and even to introduce preventive measures for potential future home confinements, as proposed by the Chinese government (30). We believe it is of great interest to examine the consequences of the recent lockdown through follow-up of the child population during the post-pandemic period in the long term.

As potential limitations of the study, it should be mentioned that since this was an online questionnaire and there was no statistical sampling, the sample may not accurately represent the general pediatric population due to possible selection bias, although the large sample size may reflect a wide variability in the population. Moreover, the cross-sectional design prevents establishing causal relationships. Another limitation is that the scales used have not been validated in the pediatric population. The authors did not believe that in the situation of strict pandemic confinement, the anxiety, depression or irritability scales were appropriate, since they were validated in a context other than strict pediatric confinement. The questionnaire was developed by an *ad hoc* panel of experts. Based on information from other studies (33, 35–41), and the opinion of the expert panel, the different dimensions were constructed to obtain a questionnaire that would meet the objective of our study in this particular context. Thus, the “emotional state” scale was created from the emotions reported by the parents to each of the questions in a dichotomous manner, which allows for simplicity in the responses of the parents since the survey was online, but it can be a limitation because it does not collect response gradient as a Likert scale does. And so, the yes/no dichotomy can lead to a loss of information.

As strengths, we highlight the large sample size and that the data collection was carried out at the very end of the lockdown, which limits recall bias and focuses responses on the real experiences of the families during the lockdown.

## Conclusions

During home confinement in Spain, an elevated percentage of children experienced important emotional effects as perceived by their parents. The factors associated with greater or lesser emotional impact on children during strict home confinement that should be taken into account to reduce these negative effects in future confinements are described.

## Data availability statement

The original contributions presented in the study are included in the article/supplementary material, further inquiries can be directed to the corresponding author/s.

## Ethics statement

The studies involving human participants were reviewed and approved by the San Juan de Alicante University Hospital Ethics Committee. Verbal consent was obtained from the participants legal guardian/next of kin. Written informed consent from the participants legal guardian/next of kin was not required to participate in this study in accordance with the national legislation and the institutional requirements.

## Author contributions

EC-G, JQ, CG-M, AN-R, FS, and EC-C: conception and design of the study, data collection, analysis, interpretation, drafting and critical revision of the manuscript, with important intellectual contributions, and approval of the final draft for publication. All authors having revised and discussed the manuscript, take responsibility, and serve as guarantors for the accuracy and integrity of the report.

## Conflict of interest

The authors declare that the research was conducted in the absence of any commercial or financial relationships that could be construed as a potential conflict of interest.

## Publisher's note

All claims expressed in this article are solely those of the authors and do not necessarily represent those of their affiliated organizations, or those of the publisher, the editors and the reviewers. Any product that may be evaluated in this article, or claim that may be made by its manufacturer, is not guaranteed or endorsed by the publisher.
