# Supplementary material for: Emotional impact on children during home confinement in Spain
Source: Front Public Health. 2022 Oct 14;10:969922. doi: 10.3389/fpubh.2022.969922 (PMC9614421; doi:10.3389/fpubh.2022.969922)
Supplement: Supplementary file 1 [file Data_Sheet_1.PDF]

Table S1. Complete descriptive analysis of all the variables analyzed

	DESCRIPTIVE	n	
Emotional state	0-2 negative emotions	994	66.2
	3-4 negative emotions	507	33.8
Sex	Male	772	51.4
	Female	729	48.6
Autonomous Community group	Other Autonomous Communities	241	16.1
	Valencian Community	1260	83.9
Only child	No	1124	74.9
	Yes	377	25.1
Location of home	No answer	137	9.1
	Urban	1167	77.7
	Rural	197	13.1
Size of home	No answer	137	9.1
	Less than 60	50	3.3
	From 60 to 120	882	58.8
	More than 120	432	28.8
Outdoor space at home (garden or terrace)	No answer	137	9.1
	Yes	697	46.4
	No	667	44.4
Underlying disease	Yes	161	10.7
	No	1340	89.3
Parents are Healthcare workers	Yes	338	22.5
	No	1163	77.5
Security	Yes	70	4.7
	No	1431	93
Other essential work	Yes	590	39.3
	No	911	60.7
Have had COVID-19	No answer	891	59.4
	No	512	34.1
	Yes, but no one in our household	70	4.7
	Yes, someone in our household	28	1.9
Pets in the home	Yes	610	40.6
	No	891	59.4
Disability	Yes	37	2.5
	No	1464	97.5

	**Dimensions: Communication, Normality after the pandemic, Disease control and Non-emotional involvement**	n	
Information given to the children	I have tried to be honest, including the most negative aspects	1427	95.1
	I have tried to be honest but avoiding the most negative aspects	74	4.9
	I have preferred not to give information	1276	85.0
Questions about the pandemic	Yes	576	38.4
	No	1066	71.0
Type of information given to the children	Realistic information	284	18.9
	Information misrepresenting the negative aspects	74	4.9
	Creative explanations	77	5.1
	No information	1396	93.0
Information adapted to the age of the children	Yes	48	3.2
	No	57	3.8
	I'm not sure	593	39.5
Sleep disturbance	Yes	908	60.5
	No	356	23.7
Changes in appetite	Has more	896	59.7
	No change	249	16.6
	Has less	281	18.7
Changes in diet	Better	926	61.7
	No change	294	19.6
	Worse	994	66.2
Hours of screen time (children)	No answer	1372	91.4
	Does not use	1	0.1
	<1h	7	0.5
	1-2h	24	1.6
	2-3h	45	3.0
	3-4h	26	1.7
	>4h	26	1.7
Medical consultation	Yes	264	17.6
	No	1237	82.4
Return to activity after pandemic	Yes	509	33.9
	No	992	66.1

Table S2. Quantitative variables in the descriptive analysis

	n	Minimum	Maximum	Mean	SD
Age	1501	0	16	6.78	3.24
Number of children in family	1501	1	10	1.93	0.78
Number of adults	1501	1	6	2.03	0.48

Table S3. Complete bivariate analysis of the variables analyzed for emotional state (good: 0-2 negative emotions or poor: 3-4 negative emotions, including sadness, fear, irritability and physical symptoms)

		0-2 negative emotions		3-4 negative emotions		
		n	%	n	%	p-value
Information adapted to the age of the children	I have tried to be honest	931	65.2%	496	34.8%	<0.001
	I have preferred not to give information	63	85.1%	11	14.9%	
Situation will normalize after the pandemic	Yes	922	72.3%	354	27.7%	<0.001
	No	72	32.0%	153	68.0%	
Underlying disease	Yes	93	57.8%	68	42.2%	0.016
	No	901	67.2%	439	32.8%	
Autonomous Community (regions)	Other Autonomous Communities	170	70.5%	71	29.5%	0.122
	Valencian Community	824	65.4%	436	34.6%	
Sex	Male	528	68.4%	244	31.6%	0.067
	Female	466	63.9%	263	36.1%	
Only child	No	719	64.0%	405	36.0%	0.001
	Yes	275	72.9%	102	27.1%	
Parents healthcare workers	Yes	235	69.5%	103	30.5%	0.145
	No	759	65.3%	404	34.7%	
Security		52	74.3%	18	25.7%	0.144
		942	65.8%	489	34.2%	
Other essential work	Yes	375	63.6%	215	36.4%	0.079
	No	619	67.9%	292	32.1%	
Pets in the home	Yes	419	68.7%	191	31.3%	0.095
	No	575	64.5%	316	35.5%	
Questions	Yes	509	55.0%	416	45.0%	<0.001

		0-2 negative emotions		3-4 negative emotions		
		n	%	n	%	p-value
about the pandemic	No	485	84.2%	91	15.8%	
Type of information given to the child	Realistic information	695	65.2%	371	34.8%	<0.001
	Information misrepresenting the negative aspects	172	60.6%	112	39.4%	
	Creative explanations	62	83.8%	12	16.2%	
	No information	65	84.4%	12	15.6%	
Information adapted to the age of the child	Yes	922	66.0%	474	34.0%	0.012
	No	40	83.3%	8	16.7%	
	I'm not sure	32	56.1%	25	43.9%	
Sleep disturbances	Yes	284	47.9%	309	52.1%	<0.001
	No	710	78.2%	198	21.8%	
Changes in appetite	Has more	205	57.6%	151	42.4%	<0.001
	No changes	675	75.3%	221	24.7%	
	Has less	114	45.8%	135	54.2%	
Changes in diet	Better	181	64.4%	100	35.6%	<0.001
	No changes	649	70.1%	277	29.9%	
	Worse	164	55.8%	130	44.2%	
Children's screen time	No answer	912	66.5%	460	33.5%	0.785
	Does not use	1	100.0%	0	0.0%	
	<1h	5	71.4%	2	28.6%	
	1-2h	14	58.3%	10	41.7%	
	2-3h	30	66.7%	15	33.3%	
	3-4h	18	69.2%	8	30.8%	
	>4h	14	53.8%	12	46.2%	
Medical consultation	Yes	153	58.0%	111	42.0%	0.002
	No	841	68.0%	396	32.0%	
Return to activity after pandemic	Yes	261	51.3%	248	48.7%	<0.001
	No	733	73.9%	259	26.1%	
Have had COVID-19	No answer	598	67.1%	293	32.9%	0.186
	No	339	66.2%	173	33.8%	
	Yes. but no one in our household	38	54.3%	32	45.7%	
	Yes. someone in our household	19	67.9%	9	32.1%	
Location of home	No answer	88	64.2%	49	35.8%	
	Urban	767	65.7%	400	34.3%	0.363
	Rural	139	70.6%	58	29.4%	
Size of home	No answer	88	64.2%	49	35.8%	

		0-2 negative emotions		3-4 negative emotions		
		n	%	n	%	p-value
	Less than 60	27	54.0%	23	46.0%	0.031
	From 60 to 120	572	64.9%	310	35.1%	
	More than 120	307	71.1%	125	28.9%	
Outdoor space at home (garden or terrace)	No answer	88	64.2%	49	35.8%	
	Yes	501	71.9%	196	28.1%	<0.001
	No	405	60.7%	262	39.3%	
Disability	Yes	19	51.4%	18	48.6%	0.053
	No	975	66.6%	489	33.4%	

Table S4. Quantitative variables in the bivariate analysis of emotional state

		n	Mean	SD	p-value
Age	0-2 negative emotions	994	6.55	3.31	<0.001
	3-4 negative emotions	507	7.23	3.05	
Number of children in the family	0-2 negative emotions	994	1.90	0.79	0.045
	3-4 negative emotions	507	1.99	0.76	
Number of adults	0-2 negative emotions	994	2.04	0.50	0.376
	3-4 negative emotions	507	2.02	0.44	
